# High-dimensional therapeutic inference in the focally damaged human brain

**DOI:** 10.1093/brain/awx288

**Published:** 2017-11-15

**Authors:** Tianbo Xu, Hans Rolf Jäger, Masud Husain, Geraint Rees, Parashkev Nachev

**Affiliations:** 1 Institute of Neurology, UCL, London, WC1N 3BG, UK; 2 Department of Clinical Neurology, University of Oxford, Oxford OX3 9DU, UK; 3 Institute of Cognitive Neuroscience, UCL, London WC1N 3AR, UK; 4 Faculty of Life Sciences, UCL, London, WC1E 6BT, UK; 5 Wellcome Trust Centre for Neuroimaging, UCL, London WC1N 3BG, UK

**Keywords:** therapeutic inference, focal brain injury, neuroimaging, stroke, neuroanatomy

## Abstract

See Thiebaut de Schotten and Foulon (doi:10.1093/brain/awx332) for a scientific commentary on this article.

Though consistency across the population renders the extraordinarily complex functional anatomy of the human brain surveyable, the inverse inference—from common functional maps to individual behaviour—is constrained by marked individual deviation from the population mean. Such inference is fundamental to the evaluation of therapeutic interventions in focal brain injury, where the impact of an induced structural change in the brain is quantified by its behavioural consequences, inevitably refracted through the lens of lesion-outcome relations. Current therapeutic evaluations do not incorporate inferences to the individual outcome derived from a detailed specification of the lesion anatomy, relying only on reductive parameters such as lesion volume and crudely discretised location. Examining 1172 patients with anatomically registered focal brain lesions, here we show that such low-dimensional models are highly insensitive to therapeutic effects. In contrast, high-dimensional models supported by machine learning dramatically improve sensitivity by leveraging complex individuating patterns in the functional architecture of the brain. The failure to replicate in humans positive interventional effects in experimental animals is thus revealed to have a remediable inferential cause, forcing a radical re-evaluation of therapeutic inference in the human brain.

## Teaser

Studying 1172 patients with anatomically registered focal brain lesions, Xu *et al.* show that high-dimensional modelling of the focally damaged human brain reveals substantial therapeutic effects opaque to current low-dimensional inferential approaches, forcing a re-evaluation of current therapeutic inference.

## Introduction

To establish a causal relation between a therapeutic intervention and its outcome requires cognisance of all the biological factors on which the effect of the intervention depends, and from which it must be isolated. In the context of focal brain damage, this relation is determined by a wide ‘causal field’ (Mackie, 1974) of many biological factors, predominantly reflecting the interaction between two distinct, complex anatomical patterns: the distribution of focal damage (Mah *et al.*, 2014*a*), and the distribution of the underlying functional anatomy (Sporns, 2011; Glasser *et al.*, 2016). For example, a single focus of damage involving only inessential neural loci may lead to spontaneous recovery, while at the other extreme, a multifocal pattern involving all critical neural loci may lead to an irreversible deficit. Such complexity inevitably requires many parameters to describe: a ‘high-dimensional’, multivariate model of the functional anatomy and its perturbation by brain injury.

Instead of modelling the full causal field, conventional therapeutic studies commonly ignore it, treating the wide variation in individual outcomes it introduces as noise. Using low-dimensional multivariate models with just a few variables, they typically include only crude, global anatomical factors such as the volume of damaged tissue, assuming the mass of the brain to be anatomically interchangeable (Kidwell *et al.*, 2001). Such models are fundamentally underparameterized, only weakly capable of isolating an intervention from the causal field in which it is embedded. The surprisingly common failure to replicate in humans substantial therapeutic effects observed in anatomically simpler animals (Gladstone *et al.*, 2002) could thus be an artefact of differences in functional–anatomical complexity, not necessarily any real differences in physiology. Though the physical limits of our clinical investigations will render some causal factors inaccessible, the obstacles to modelling the wealth of anatomical factors provided by routine neuroimaging—likely to be causally the most important—are merely inferential.

## Materials and methods

**Figure 1:**
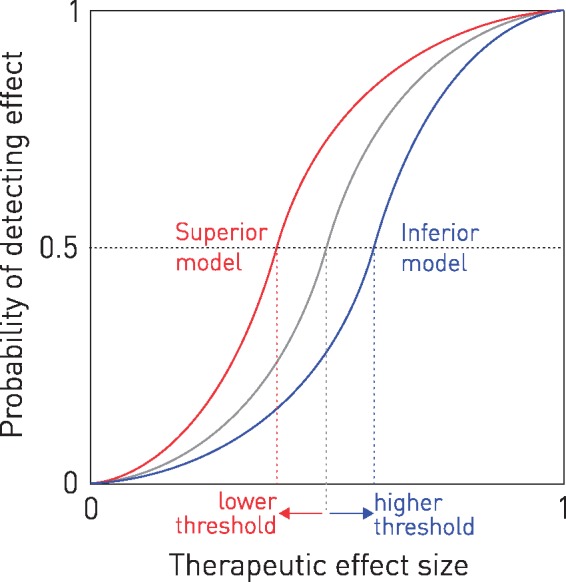
**Therapeutic functions.** The relation between a given therapeutic effect size and the probability of correctly detecting it in a set of trials of the intervention is described by a continuous monotonic function across the range of 0 (no effect) to 1 (all treated patients respond). The midpoint of this function is the point where half of all trials yield a positive result, i.e. where a meta-analysis will only just identify the intervention as successful. The corresponding point on the abscissa is the threshold: the minimum effect size required to identify the intervention as successful. This threshold—a synoptic index of the detectability of the intervention—will be shifted to the right if the inferential model is less able to remove variability that obscures the therapeutic effect (in blue), and to the left it is more able (in red). See Supplementary material.

To quantify the impact of this problem we must evaluate a set of hypothetical interventions against a set of real data, directly comparing low- and high-dimensional inferential methods. The intervention must be hypothetical because we cannot distinguish between an inadequate model and an insufficient effect where the therapeutic effect is, by definition, unknown. We must instead evaluate a comprehensive range of possible effect sizes so as to determine the therapeutic threshold: the minimal effect the inferential method is able confidently to detect. By iteratively randomizing the data into ‘intervention’ and ‘control’ groups, and simulating interventions of varying effect size, we can derive a continuous, empirical therapeutic function describing the relation between the size of an interventional effect and the probability of detecting it with a given inferential method (Fig. 1). The steeper and closer the function is to the left, the more sensitive the method. Performing many randomized evaluations per point—essentially simulating a large scale meta-analysis of many studies—allows us to calculate confidence intervals for each computed function, formalizing the comparison of inferential performance.

Though interventions deployed in the context of focal brain damage are mechanistically diverse, they fall into two cardinal classes: ‘lesion-altering interventions’ that change the characteristics of the lesion itself, e.g. thrombolytic therapy in stroke (Wardlaw *et al.*, 1997); and ‘lesion non-altering interventions’ that change the brain’s response to it, e.g. neurorehabilitation (Johansson, 2000). Interventions of the former kind reduce the volume of the lesion, and can be hypothetically modelled by artificially shrinking it. Interventions of the latter kind alter the clinical outcome directly, and can be hypothetically modelled by varying the proportion of treatment ‘responders’. Both classes critically depend on the impact of the lesion on the underlying functional anatomy, but the former is theoretically more sensitive as it definitionally involves a therapeutically induced anatomical change (Supplementary material).

We need a set of hypothetical interventions to traverse the full range of therapeutic effect sizes, but we need real data, on a scale large enough to make the underlying complexity tractable, to quantify the real-world benefit of adopting one inferential method over another. Exploiting previously validated automatic segmentation of clinical MRI for stroke, here we assembled a large set of focal human brain lesion data (*n* = 1172), non-linearly registered in standard stereotactic space so as to allow direct comparisons across individuals with high anatomical fidelity (Mah *et al.*, 2014*a*, *b*). The processed images indexed the presence or absence of acute damage at each anatomical location across the entire brain with a resolution of 6 mm^3^, yielding 5789 binary variables per patient: a rich, high-resolution parameterization of both lesion and brain anatomy. We chose acute stroke, imaged with a diffusion-weighted MRI sequence reliably sensitive to ischaemic injury, as the most prevalent cause of focal brain dysfunction (Feigin *et al.*, 2014) (Supplementary material).

The primary measure of therapy in the brain is the resultant behaviour: the functional impact of the biological change the therapy induces. Here we chose a key aspect of behaviour—the preferred direction of gaze at rest—as this could be objectively and contemporaneously determined from automated eye lens segmentation of the brain imaging itself (Becker and Karnath, 2010). Deviations of gaze reflect disruption of fundamental oculomotor and attentional neural circuits, often (but not always) recover following brain injury, and are essential to the description of the patient’s neurological state (Ramat *et al.*, 2007). By obtaining the direction of gaze at two time points—on the admission CT scan, and on the subsequent MRI scan typically 24 to 72 h later—we could identify dynamic changes in this specific behavioural parameter over time, and its relationship to brain anatomy. Each high-dimensional lesion pattern was thus coupled to an objectively quantified degree of gaze deviation, at the precise point of imaging, including the temporal trajectory of any recovery in the critical early period of a stroke (Supplementary material).

This dataset now allowed us to quantify the impact of underparameterization on therapeutic inference by comparing the probability of detecting a hypothetical interventional effect within low- and high-dimensional models of the same data, iteratively resampled into randomized cohorts of ‘intervention’ and ‘control’ groups. In a conventional study, an intervention is evaluated against the behavioural outcome, deconfounded by a small set of demographic and disease-specific factors, with the imaging appearances modelled by few global parameters such as lesion volume. In the high-dimensional approach we explore here, an intervention is evaluated in exactly the same way except that the imaging parameters are expanded to the many we argue are necessary adequately to model the interaction between lesion patterns and functional anatomy.

## Results

**Figure 2:**
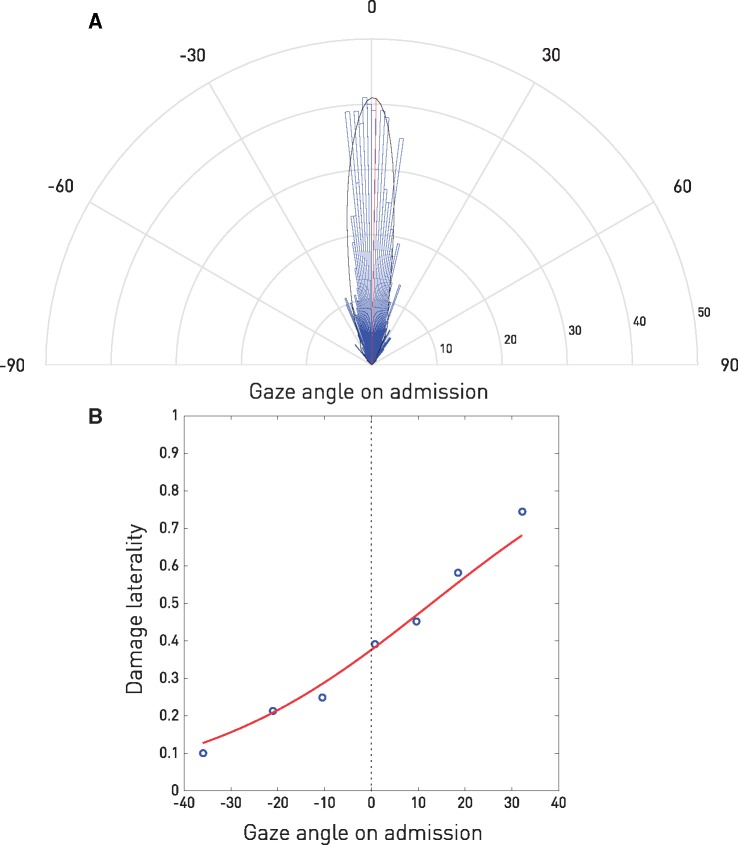
**Distribution of patient gaze on admission.** (**A**) Polar plot of the histogram (blue) and kernel density estimate (black) of the distribution of patient gaze on admission as determined by semi-automated segmentation of the intraocular lenses visualized on the CT scan. Note the circular mean (red) is within <1° of the midline (0.93°). (**B**) Relation between the admission direction of gaze and the laterality of brain damage. For each of seven bins of gaze angle, the mean ratio of the volume of right hemisphere damage to the overall volume of damage is plotted (blue), with a general linear model maximum likelihood fit of the relation across gaze (red). The remarkably strong dependence of gaze on damage laterality shows the variation in gaze shown in **A** is unlikely to be dominated by noise. See Supplementary material.

The population distribution of gaze at each time point was centred on the midline, but was strongly biased by the lesion, demonstrating its dependence on the underlying anatomy and its suitability for modelling therapeutic inference (Fig. 2).

First, we focused on modelling lesion non-altering interventions such as neurorehabilitation that hypothetically increased the proportion of patients recovering from a deviated to a normal gaze between the two time points without altering the lesion itself. We evaluated a series of models where a hypothetical intervention iteratively randomized across a sample of 1172 patients led to ‘recovery’ of the gaze of a randomly-selected proportion of those treated, varying from 10% to 90% recovery in separate models. Abnormal gaze was defined as a leftward deviation of >12° at the first time-point, ‘recovery’, as a deviation within 3° of the midline at the second time point.

**Figure 3:**
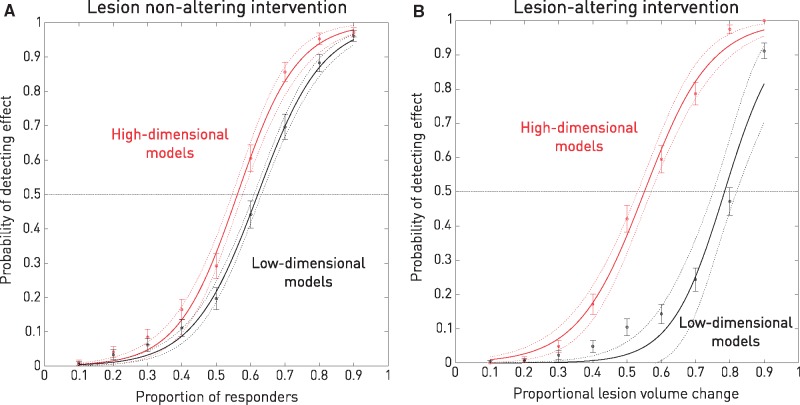
**Empirical therapeutic inference functions.** (**A**) Lesion non-altering interventions. For a set of hypothetical lesion non-altering interventions that normalized gaze in a proportion of those treated varying from 0.1 to 0.9, the mean probability of detecting an intervention was determined from 600 iterative randomizations per treatment level with two different kinds of models. For the low-dimensional approach (black), linear regression models of the data incorporated only the factors of intervention, age, sex, and lesion volume, labelling each ‘trial’ as positive if the *P*-value for the intervention was <0.05. The error bars correspond to 95% CI of the means. A continuous function was fitted to the mean data using a robust spline fit, with estimates of 95% CI given in dotted lines. For the high-dimensional approach (red), linear regression models of the data and subsequent analysis were identical except for adding a high-dimensional predictor of the gaze outcome regardless of any treatment. Note that the high-dimensional approach substantially shifts the threshold of the therapeutic function to the left, reflecting enhanced sensitivity for detecting a therapeutic effect. (**B**) Therapeutic inference for lesion-altering interventions: For a set of hypothetical lesion-altering interventions that reduced the volume of the lesion by 0.1 to 0.9, the mean probability of detecting an intervention was determined from 600 iterative randomizations per treatment level with two different kinds of models. For the low-dimensional approach (black), linear regression models of the data incorporated only the factors of intervention, age, sex, and pretreatment lesion volume, labelling each ‘trial’ as positive if the *P*-value for the intervention was <0.05. The error bars correspond to 95% CI of the means. A continuous function was fitted to the mean data using a robust spline fit, with estimates of 95% CI given in dotted lines. For the high-dimensional approach (red), the linear regression models of the data and subsequent analysis were identical except for adding a high-dimensional predictor of the gaze outcome regardless of any treatment. Note that the high-dimensional approach substantially shifts the threshold of the therapeutic function to the left, reflecting enhanced sensitivity for detecting a therapeutic effect, even more so than for lesion non-altering interventions. See Supplementary material.

Following standard practice in interventional studies, we fitted low-dimensional multivariate linear regression models to the data that incorporated the factors of intervention, age, sex, and lesion volume, predicting recovery as the dependent variable. The significance value for the factor of intervention, set at *P* < 0.05, determined if the ‘trial’ was considered positive or negative. Iterating over 600 randomizations for each of nine effect sizes ranging from 10% to 90% response, this analysis yielded a monotonic therapeutic function with a therapeutic threshold corresponding to 62.9% [confidence interval (CI) = 61.5–63.4%] of those treated responding to the intervention (Fig. 3A, in black). The conventional approach to evaluating an intervention, examined as a large scale meta-analysis does, is thus shown to be remarkably insensitive.

**Figure 4:**
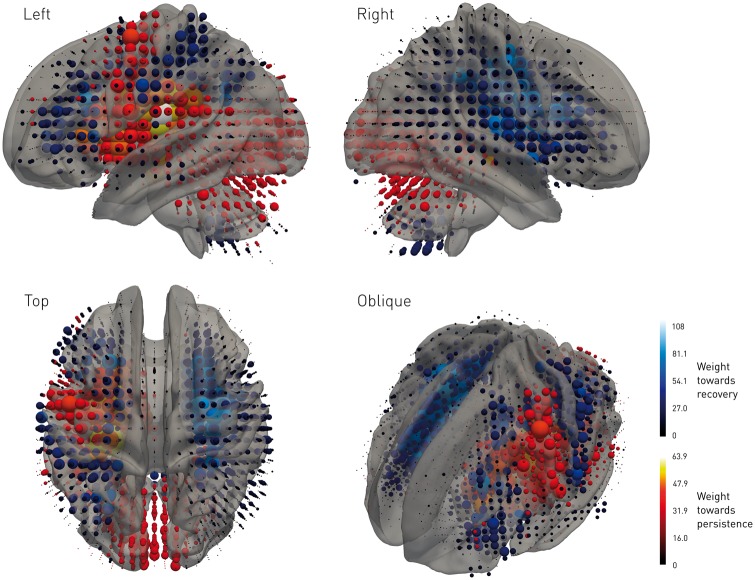
**Gaze recovery high-dimensional classifier weights.** Represented as 3D cubic glyphs varying in colour and scale are the weights of a transductive linear support vector machine classifier trained to relate the high-dimensional pattern of damage to gaze outcome, achieving *k*-fold cross-validation performance of 78.33% (SE = 1.70%) sensitivity and 82.78% (SE = 0.56%) specificity for distinguishing between patients who recovered from a leftward deviation of gaze and those who did not. Positive weights (dark blue to cyan) favour recovery, negative weights (dark red to yellow) persistence of symptoms. Though hemispheric asymmetry is prominent, note the distribution of weights is highly complex, as one would expect from the complexity of the functional and lesional architectures that generate the critical pattern. See Supplementary material.

A high-dimensional model where the pattern of focal damage is adequately parameterized should detect an intervention effect more accurately by taking into account those who would naturally recover without any treatment. To quantify the impact of this theoretical advantage, we ran a set of modelled interventions with exactly the same parameters as before except that to the standard linear regression model was added an additional covariate factor predicting whether or not the patient would recover regardless of any intervention. This factor was estimated from a multivariate classifier, here a transductive linear support vector machine, trained to relate the high-dimensional anatomical pattern of damage to the gaze outcome (Fig. 4) (Sindhwani and Keerthi, 2006). Crucially, this classifier was trained on wholly independent samples of the data, and received no information whatsoever on the randomization of the intervention in each model. Its contribution was thus limited to accounting for a proportion of the natural outcome variance explained by the pattern of damage that would otherwise contaminate the critical contrast of intervention. The classifier’s performance on test data [78.33%, standard error (SE) = 1.70% sensitivity and 82.78% (SE = 0.56%) specificity] reflected substantial power to capture this. This dimensionality enhancement substantially improved therapeutic inference, significantly shifting the therapeutic function leftward to 56.0% (CI = 54.65–57.35%) (Fig. 3A, in red). As predicted, reanalysing the same data within a high-dimensional framework potentially enables us to detect the value of interventions that would otherwise be erroneously discounted as ineffective.

Where the lesion does not change, knowledge of the relation between the pattern of injury and the outcome increases sensitivity by identifying the patients who would recover anyway. But with a lesion-altering intervention such as thrombolysis*,* the value of such knowledge is amplified because the impact of a change in the anatomy of the lesion can now be directly linked to the outcome. To take extreme cases, a large lesion centred on a small, discrete critical neural locus must shrink a great deal for the outcome to improve; a small lesion close to the edge of more diffuse critical neural focus need only shrink minimally to produce the same effect. A lesion-altering intervention that produces exactly the same change in lesion volume can thus have a diversity of behavioural consequences, entirely dependent on the intersection of lesion and functional anatomy only a high-dimensional method can plausibly capture. If volume of brain saved—the defining action of the intervention—is the physiological effect but behavioural outcome the measure, low-dimensional therapeutic inference will be only poorly sensitive.

To examine this second scenario, we constructed a series of randomized models where a hypothetical intervention induced a range of 10% to 90% lesion volume reduction, simulated through iterative surface erosion of the ‘treated’ lesion maps. The critical outcome remained the behaviour—resolution or persistence of the deviation of gaze. If the relation between the pattern of damage and outcome is predictable across individuals—as clearly demonstrated by our gaze-trained classifier—then it must be at least as predictable in the same individual where the lesion pattern changes. The outcome following the intervention in each modelled case was thus determined by the voxel-wise weights of the previously trained classifier—now applied to each changed lesion—as the best evidence for what would have happened had the lesion changed in reality (Fig. 4). As before, we then fitted standard linear regression models to the outcome data that incorporated factors of intervention, age, sex, and pre-intervention lesion volume, labelling the ‘trial’ as positive if the *P*-value for the intervention was <0.05. Iterating over 600 sets of randomizations for each of nine effect sizes ranging from 10% to 90% volume reduction, this analysis yielded a therapeutic function with a threshold at an effect size of 78.4% (CI = 75.75–81.05%) lesion volume saved (Fig. 3B, in black). Even more prominently than with lesion non-altering interventions, a very substantial effect size was necessary reliably to identify the intervention as successful.

To quantify the benefit of high-dimensional modelling here, we re-ran the same models with an additional covariate factor capturing the relation between the pre-intervention lesion pattern and gaze outcome with a transductive linear support vector machine. As before, this factor contained no information about the intervention itself. The therapeutic function shifted leftward to 55.0% (CI = 53.1–56.9%): as predicted, a greater gain than for lesion non-altering interventions, and even stronger motivation for adopting the high-dimensional approach (Fig. 3B, in red).

## Discussion

These analyses demonstrate empirically an analytic truth: where a multiplicity of interacting factors—a causal field—determine an outcome, isolating the contribution of any single factor, such as an intervention, leans heavily on our knowledge of all the others. Where such collateral factors are numerous and complex in their interactions, i.e. the system is high-dimensional, as has been abundantly demonstrated of the human brain, it may be tempting to treat them as the noise they superficially resemble. Indeed, without the recent rise in the inferential power of machine learning, we had no other practicable option, and the consequent critical underparameterization was impossible either to prove or disprove. But now that the complexity has been shown to be tractable, therapeutic inference in the brain may be dramatically enhanced, given data of sufficient scale to constrain the vast space of possible solutions high dimensionality inevitably opens, and an inferential framework tailored to its demands.

It is crucial to recognize that high-dimensional modelling does not merely identify a small minority of idiosyncratic cases for whom the intervention exceptionally works: this is not ‘subgroup analysis’. Rather, it properly accounts for the numerous anatomical factors that influence the outcome in each and every case, exactly as a set of conventionally modelled confounds does, but vastly expanded, so that the specific effect of the intervention can be properly isolated. Our data and simulations suggest that this accounting for different anatomical factors is critically important to evaluating the effect of an intervention with high sensitivity. Just as age—a near-universal confound—is always modelled, so should the full causal field of material factors, however complex it might be. And we know for certain that the functional anatomy of the brain is both complex and material to the outcome where injury to the brain is focal.

Equally, our approach does not overpower materially insignificant effects into statistical significance, for we have seen that conventional therapeutic inference is insensitive to effects of substantial size, as is suggested by the translational failure of so many agents potent in experimental animals. Nor is the functional visibility of an interventional effect critical here: if a behavioural measure is poorly related to volume of brain saved, the measure is more likely to be weak than the brain truly unnecessary. That we do not, for example, have any routine measures for the function of the frontal pole—arguably the most distinctively human part of the brain—does not mean its integrity is inconsequential (Burgess *et al.*, 2007).

Though the benefit from high-dimensional modelling is bound to vary with the behavioural outcome studied, it can only be greater the more distributed the underlying critical functional architecture. Since the complexity of neural systems controlling gaze will likely be exceeded by many others, our estimates are likely to be conservative. Indeed, the deepening appreciation of the fundamentally interconnected nature of the brain points decisively towards a need for greater, not lesser, complexity in our explanatory models, not just of functional anatomy (Bzdok and Yeo, 2017; Eickhoff *et al.*, 2017), but of behaviour itself (Krakauer *et al.*, 2017). Therapeutic inference must inevitably reflect this; moreover, the payoff is not only a greater understanding of the brain but potentially the correct appreciation of many treatments hitherto erroneously thought to be ineffective.

## Supplementary Material

Supplementary Figures
