# Supplementary material for: High-dimensional therapeutic inference in the focally damaged human brain
Source: Brain. 2017 Nov 15;141(1):48–54. doi: 10.1093/brain/awx288 (PMC5837627; doi:10.1093/brain/awx288)
Supplement: Supplementary Figures [file brain-2017-01065-file006_awx288.pdf]

Supplementary Material: Detailed Methods

Patients

A set of 1172 patients admitted to the University College London Hospitals (UCLH) acute stroke service over the past decade was identified where the clinical diagnosis was acute ischaemic stroke, computed tomography (CT) and diffusion weighted magnetic resonance imaging (DWI) had both been performed, and at least one definite acute ischaemic lesion could be identified on the DWI. CT is routinely performed on all patients suspected of acute stroke at UCLH; MR on the majority, constrained mostly by contraindications and tolerability. So as to reduce bias from the global effects of very large lesions, only patients with DWI-quantified lesion volume of less than 50 mls were included: ~90th centile for our clinical population. The sample is therefore reasonably representative of acute stroke.

The age distribution ranged from 18 to 97 years with a mean of 63.89 and a standard deviation of 15.91. The proportion of males was 0.561 (Supplementary Fig. 1).

Ethnicity was disclosed in the clinical record for 952 of the 1172 patients (Supplementary Fig. 2). The distribution reflects the constitution of the hospital's natural catchment area.

Imaging

All patients underwent both CT and MR imaging.

CT data acquisition

For each patient, either a Somatom Definition or a Somatom Sensation (both Siemens, Erlangen, Germany) were used to acquire an uncontrasted whole head image of typical resolution 0.48 mm x 0.48 mm x 5 mm. This acquisition was done in the course of routine clinical care, typically within a few hours of admission to hospital. The image was reported by a neuro-radiologist, and contributed to the final diagnosis on which recruitment depended as defined above, generally by helping eliminate alternative diagnoses such as primary intracerebral haemorrhage.

CT data analysis

Each CT image was rigidly co-registered to the standard SPM12 tissue probability map in MNI space using normalised mutual information with adjustment from a Procrustes analysis weighted by the white and grey matter compartments (Jha et al., 2016). The degree of head yaw estimated by the procedure was recorded. The axial slices in which each intraocular lens was best seen—i.e. where the slice passed closest to the lens's centroid—were manually identified by a trained operator (PN), and each lens was semi-manually segmented with the aid of MIPAV's levelset VOI tool (https://mipav.cit.nih.gov/) on its corresponding (usually the same or immediately neighbouring) slice. Both natural

and prosthetic lenses were easily segmentable in this manner. In the small minority of patients where one lens was either not visible or absent, only one lens was segmented. The voxel coordinates of each lens boundary were then used to generate a binary mask of the lens with the aid of Matlab's *poly2mask* function and thereafter to derive the centroid of each mask and its orientation with the aid of Matlab's *regionprops* function (https://uk.mathworks.com/products/matlab.html). The coordinates of the centroid allowed us automatically to determine the side of the corresponding eye; the orientation to determine the direction of gaze for each eye in the axial plane relative to the head. In combination with the estimate of head yaw from the image registration procedure we could then derive the direction of gaze relative to the axis of the body.

MR data acquisition

All acquisitions were performed on scanners manufactured by General Electric (Genesis Signa), Philips (Achieva and Ingenia), or Siemens (Avanto, Skyra and Verio) with field strength of either 1.5 or 3 Tesla. This diversity reflects changes in routine clinical practice over the period of data collection rather than differences in individual indications. All scans were obtained as part of the clinical routine, employing clinical protocols.

We extracted from each imaging study the echoplanar diffusion-weighted (DWI) and T2-weighted sequences (T2), for lesion segmentation and gaze analysis, respectively. DWI is widely used to detect and locate acute ischemic lesions(Fiebach et al., 2002). In its clinical application, it consists of an image with a b value of 0 s/mm² that is relatively insensitive to acute ischaemia but shows reasonable tissue contrast, and an image with a b value of 1000 s/mm² that is sharply sensitive to ischaemia but has poor normal tissue contrast. This complementarity can be exploited to achieve both good lesion segmentation—which depends on the contrast between lesioned and normal tissue—and good brain registration—which depends on the contrast between normal tissue types. A T2 sequence, obtained in the same imaging session, was used to obtain better views of the intraocular lens than the DWI afforded.

Image pre-processing

All processing was performed within MATLAB (Mathworks, USA), following a processing pipeline built principally on SPM12 (http://www.fil.ion.ucl.ac.uk/spm/software/spm12/). After conversion from DICOM to NIfTI file format, each image was signal clamped to between 0.1% and 99.9% of the empirical signal distribution estimated with a robust kernel density method (Botev et al., 2010). The images were then denoised with an oracle-based 3D discrete cosine transform filter (Manjón et al., 2012). Each image was then rigidly co-registration to the standard SPM12 tissue probability map in MNI space using normalised mutual information with adjustment from a Procrustes analysis weighted by the white and grey matter compartments(Jha et al., 2016). This rigid procedure was naturally robust to the presence of a lesion in all sequences. Subsequent processing varied with modality as follows.

T2 PROCESSING: As for the CT scans, the degree of head yaw estimated by the preceding rigid body registration procedure was recorded. The axial slices in which each intraocular lens was best seen—i.e. where the slice passed closest to the lens's centroid—were manually identified by a trained operator (PN), and each lens was semi-manually segmented with the aid of MIPAV's levelset VOI tool (https://mipav.cit.nih.gov/) on its corresponding (usually the same or immediately neighbouring) slice. Both natural and prosthetic lenses were easily segmentable in this manner. In the small minority of patients where one lens was either not visible or absent, only one lens was segmented. The voxel coordinates of each lens boundary were then used to generate a binary mask of the lens with the aid of Matlab's *poly2mask* function (https://uk.mathworks.com/products/matlab.html) and thereafter to derive the centroid of each mask and its orientation with the aid of Matlab's *regionprops* function. The coordinates of the centroid allowed us automatically to determine the side of the corresponding eye; the orientation to determine the direction of gaze for each eye in the axial plane relative to the head. In combination with the estimate of head yaw from the image registration procedure we could then derive the direction of gaze relative to the axis of the body, exactly as for the CT scans.

DWI PROCESSING: So as to optimise the alignment between the b0 and b1000 images, the former was rigidly co-registered to the latter using SPM's standard co-registration routine with default settings. SPM12's normalisation/segmentation routine(Ashburner and Friston, 2005) was then applied to the b0 image so as to derive a deformation field describing the optimal non-linear transformation into standard Montreal Neurological Institute (MNI) stereotactic space. The parameters of the routine were set at default. This deformation field was then used to transform the b1000 image into MNI space, resliced to $2mm^3$ isotropic with 4th degree b-spline interpolation (Mah et al., 2012, 2014). Each image was manually checked against the SPM template to confirm satisfactory registration.

Lesion Segmentation

With all b1000 images in anatomical register we proceeded to segment each lesion by the voxel-wise application of our previously validated method based on the anomaly metric, *zeta* (Mah et al., 2012). We began by normalising the signal distribution of each individual image by subtracting from it the peak of the white matter signal distribution for that specific image. This was derived from a kernel density estimate (Botev et al., 2010) applied to voxels falling within a custom-made mask constructed by thresholding SPM12's white matter tissue map at a probability greater than 0.9 and excluding areas in the frontal and temporal poles commonly prone to artefact on DWI sequences. Where the radiological report identified a unilateral lesion—the vast majority—the mask was applied only to the unaffected hemisphere; where both hemispheres were affected the mask was applied to both hemispheres. Since ischaemic lesions tend to be small where bilateral, and the kernel density estimate is relatively robust to outliers, the signal normalisation was

equally effective in both cases. This normalisation step enabled us to adjust for global differences in the signal distribution across all scans.

For each image, independently for each voxel within the brain, we then calculated the zeta anomaly metric against a set of 492 reference images—processed in exactly the same way—derived from patients suspected of stroke in whom no acute lesion was reported by the reviewing radiologist. The zeta value of a voxel in the test image is given by the mean distance to the *k* nearest neighbours drawn from the anatomically homologous voxels in the unlesioned reference set, normalised by the mean distance between the *k* neighbours themselves(Mah et al., 2012). The distance measure here is simply the difference in the voxel signal. This approach makes the metric robust to badly behaved signal distributions, including non-monotonic ones differing substantially in density across voxels. The metric has only one parameter, *k,* here set at 30 based on previous work(Mah et al., 2012, 2014).

Zeta resultant voxel-wise zeta maps are real-numbered and dimensionless. In order to threshold them into the binary maps in customary use—denoting each point in the brain as being simply affected or unaffected—we applied a cluster-level thresholding procedure modelling the distribution of zeta values within each image under a generalized extreme value (GEV) distribution. For each cluster in each segmented lesion, the estimated GEV mean and variance were used to derive an adaptive threshold, as described in detail and validated elsewhere(Mah et al., 2012).

Behaviour

The preferred direction of gaze in a sensorially unbiased environment is sensitive to the normal operation of attentional and oculomotor mechanisms distributed across multiple areas of the brain(Corbetta et al., 1998). It is an important component of the neurological examination, and reflected in standard parameterisations of clinical deficits in stroke such as the widely used NIH Stroke Scale(Group, 1997; Muir et al., 1996). Though clinically obvious when marked, gaze deviation is not easy to quantify with precision, for no instrument is widely available for the purpose. Segmenting the lens of each eye, on each scan, allowed us to obtain a precise, objective and contemporaneous measure of preferred gaze direction in the axial plane of the head, within an environment—the bore of a CT or MRI scanner—that has no strong lateralising features, and is therefore free from external bias. The direction of gaze was taken as the average of the two eyes (where both eyes were present and segmentable: the vast majority). The yaw component of the brain image registration allowed us to derive two estimates of gaze: head-centric and body-centric. Since the head is generally supported in a midline-aligned position during imaging, the two estimates were highly correlated, and in preliminary analyses did not yield substantially divergent results (data not shown). We therefore used the head-centric values in all subsequent modelling. Gaze in each patient was thus parameterised as a single value at two points: the time of the CT on admission, and the time of the MRI within the subsequent 10 days.

Naturally, all patients received acute treatment as dictated by their clinical state, and this varied across the group. But all treatment that plausibly altered the anatomical properties of the lesion—e.g. thrombolysis—was always carried out *before* the MRI, from which the lesion parameters were extracted. The lesion pattern here therefore correctly captured the state of the brain at the second time point, taking into account any change in the lesion in the intervening period, whether spontaneous or treatment-induced.

To demonstrate the sensitivity of gaze to crude parameterisations of the brain lesion, we fitted, for each time point, general linear models of the relation between gaze angle (distributed in seven bins), and the laterality of the lesions in each bin as indexed by the mean of the ratio of the number of voxels in one hemisphere to the number in the other. As expected, gaze deviation was strongly modulated by the laterality of lesions at the time of admission (Supplementary Fig. 3), but less so at the later time of the MRI (Fig. 2B). This is exactly what one would predict if recovery is sensitive to the precise features of the detailed anatomy, which no crude laterality metric could adequately capture.

For the purpose of our therapeutic inference analyses (see below), we identified patients with a gaze deviation to the left of at least 12 degrees at the time of the CT. Those who had normalised to within 3 degrees of the midline by the time of the MRI were labelled as *recovered*; those whose deviation remained at least 6 degrees to the left were labelled as *persistent.* Naturally, normality can have no hard boundary, but what is required here is merely division into two illustrative categories materially differing in the degree of abnormality of their behaviour. We chose deviation to one side only to minimize the complexity of the likely neural dependents and thereby make the comparison with low-dimensional approaches more conservative.

Modelling therapeutic inference

The question we wish to answer is whether or not modelling the lesion architecture in detail commensurate with the underlying functional complexity improves therapeutic inference: the ability to detecting the effect of an intervention that changes the functional outcome.

Our first step is to create a framework for applying a simulated intervention of varying effectiveness to real data so that we may quantify our ability to detect it across a comprehensive range of effect sizes. We must use a simulated intervention because a real intervention offers no reliable ground truth; indeed the analysis would be circular, for the effect size of interventions has hitherto been estimated from the low-dimensional models we are evaluating here. Interventions in focal brain injury fall into two broad classes: *lesion-reducing* interventions that modify the anatomy of the lesion, and *lesion-retaining* interventions that assist the functional adaptation of the brain during recovery but do not change the lesion itself. The former, generally operate in the "hyperacute" period while threatened brain may still be salvaged, e.g. drug-induced or mechanical thrombolysis in

ischaemic stroke; the latter in the period after the lesion is established, e.g. drug-assisted or behavioural rehabilitation.

Lesion non-altering interventions

To simulate lesion-retaining interventions we simply need to change the outcome of a randomly chosen proportion of those treated to "cured". Since it is near-universal for some patients to recover regardless of treatment, this does not unambiguously identify those treated. By varying the proportion from 10% to 90%, we can then estimate a continuous function—what we here call a *therapeutic function*—describing the relation between the size of the effect and our ability to detect its presence (Fig. 1). In line with established practice, the criterion for successful detection is achieving a p value less than 0.05 on the statistical test used to evaluate a given intervention (see below for details). By evaluating 600 randomly sampled iterations at each effect size—analogously to performing a meta-analysis of many evaluations of the intervention—we can make our estimate robust and qualify its confidence statistically. To obtain adequate coverage of the therapeutic function we chose to evaluate the interval between 10% and 90% effect size, in steps of 10%, fitting a logistic function by maximum likelihood.

Lesion-altering interventions

To simulate lesion-reducing interventions it is not sufficient to change the outcome on a proportion of those treated, for if the behavioural effect of the intervention is dependent on the anatomy of the lesion it will change as the lesion itself changes. Rather, we must *shrink* the lesion—reasonably achieved by morphological surface erosion of the binary lesion maps—and determine the individual outcome dependent on whether or not critical functional areas thereby escape damage. The effect size then is reduction in lesion volume, expressed as percentage of the untreated lesion volume. To achieve lesion shrinkage by controllable amounts, we applied erosion iteratively to each lesion until the percentage effect size was met, generating nine versions of each lesion shrunk across the range of 10% to 90%. The therapeutic function modelling otherwise remained as above.

Low- and high-dimensional models

For each class of intervention, we need to compare the performance of two sets of models differing only in the parameterisation of the lesion. In the low-dimensional multivariate case in current use the lesion is parameterised only by its total volume, in the high-dimensional multivariate case we propose here it is parameterised by the presence or absence of damage across the entire brain for voxels, sampled at 6mm x 6mm x 6mm resolution, hit at least twice: 5789 variables overall.

A small number of variables is tractable with a standard general linear model (GLM) estimated with conventional least-squares minimization. We therefore implemented the low-dimensional models as GLMs where the response variable was recovery or persistence of left gaze deviation as defined above. The predictor variables were

treatment, age, sex, and volume of the lesion, as is broadly conventional in therapeutic studies.

A conventionally estimated GLM cannot reliably model data where the number of variables substantially exceeds the number of cases. Such overdetermined models may perform poorly not because the relevant additional variables do not improve the fit but because the irrelevant variables degrade it more. A different inferential framework is therefore required. Here we chose to employ a linear transductive support vector machine, SVM_{Lin}, (Sindhwani and Keerthi, 2006) for the following reasons. First, the foundations of kernel machines are well-established and their diverse implementations widely used in science and beyond. Second, the specific implementation here is well-suited to classification problems with large numbers of features and cases. Finally, it performs well with sparse data, such as binarized lesion maps. Third, the transductive element allows us to use unlabelled data to improve classification performance, thereby using all available data.

We used SVM_{Lin} to train a multi-switch classification model with the regularisation parameters λ and λ´, the maximum number of switches, and the positive class fraction of unlabelled data optimised by 10 times cross-validation, applied to a randomly chosen 80% of the dataset. The target variable was change in gaze—recovered vs persistent—and the predictor variables were all within-brain voxels hit two or more times within the dataset: 5789 variables. The best model achieved excellent predictive performance on the subset of the data not used in training: sensitivity 78.33% (se=1.70%) and specificity was 82.78% (se=0.56%).

To make the high- and low-dimensional models most directly comparable, we used the SVM_{Lin} classifier to generate a single regressor predicting the natural outcome in each patient—*regardless of any simulated treatment*—and added this as a covariate to a GLM that was otherwise identical to the low-dimensional case. The predictor variables here were treatment, age, sex, volume of the lesion, and the classifier-derived natural outcome predictor. Note that the outcome predictor contained *no* information about the randomisation of treatment, and so could optimise the detection of a treatment effect only by better prediction of the natural outcome.

In the case of lesion non-altering interventions, for each of the effect sizes between 10% and 90% intervention-induced recovery, we modelled 600 different randomisations of the data separately with low and high-dimensional GLMs, differing only in the inclusion of the SVM_{Lin}-estimated predictive factor. Models where the p value associated with the "treatment" factor < 0.05 were labelled as positively identifying the treatment, generating an estimate of the sensitivity of the model at that effect size. The sensitivity values at each effect size were then used to generate separate therapeutic functions for the low- and high-dimensional models, including their confidence intervals.

In the case of lesion-altering interventions, we performed the modelling in exactly the same way except that the "treatment" effect was exerted via lesion shrinkage to the specified percentage in all treated patients. For each treated image, we therefore needed

an estimate of the new outcome given the shrunken lesion. This was estimated with the SVM_{Lin} classifier, now fed a shrunken version lesion it had not previously seen. Note the outcome-predictive factor included in the GLM remained that derived from *untreated lesions*, and so plausibly accounted only for the variability resulting from natural variations in the outcome. As with the lesion non-altering intervention models, no information about the treatment was therefore conveyed in this factor.

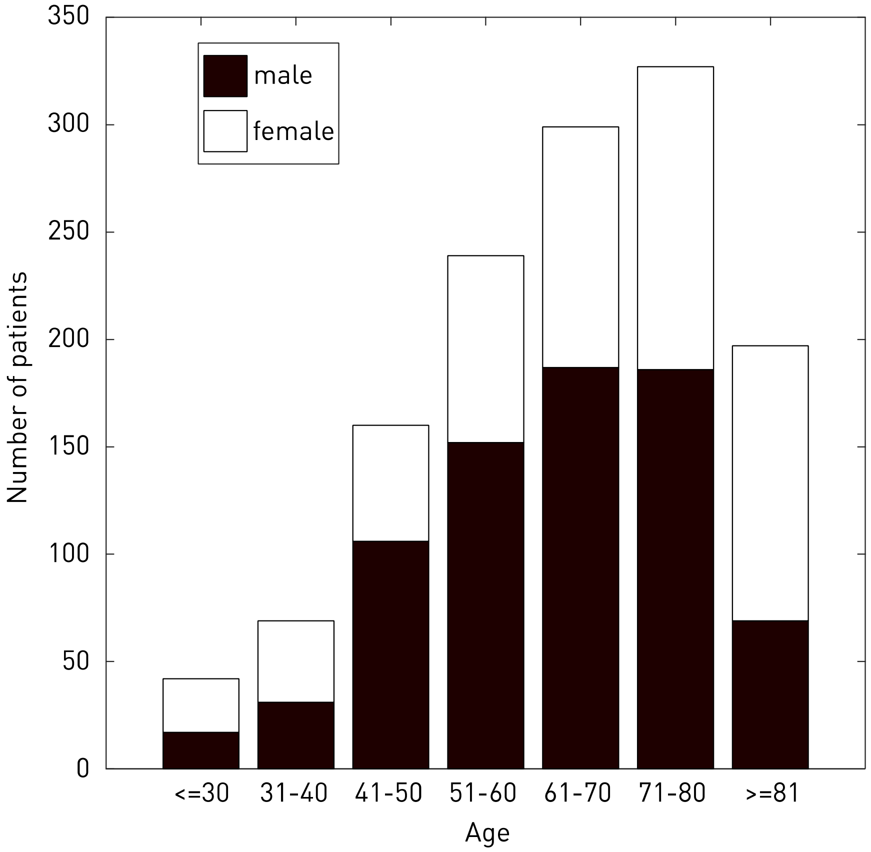

Supplementary Figure 1.

Age and sex distribution of the patients.

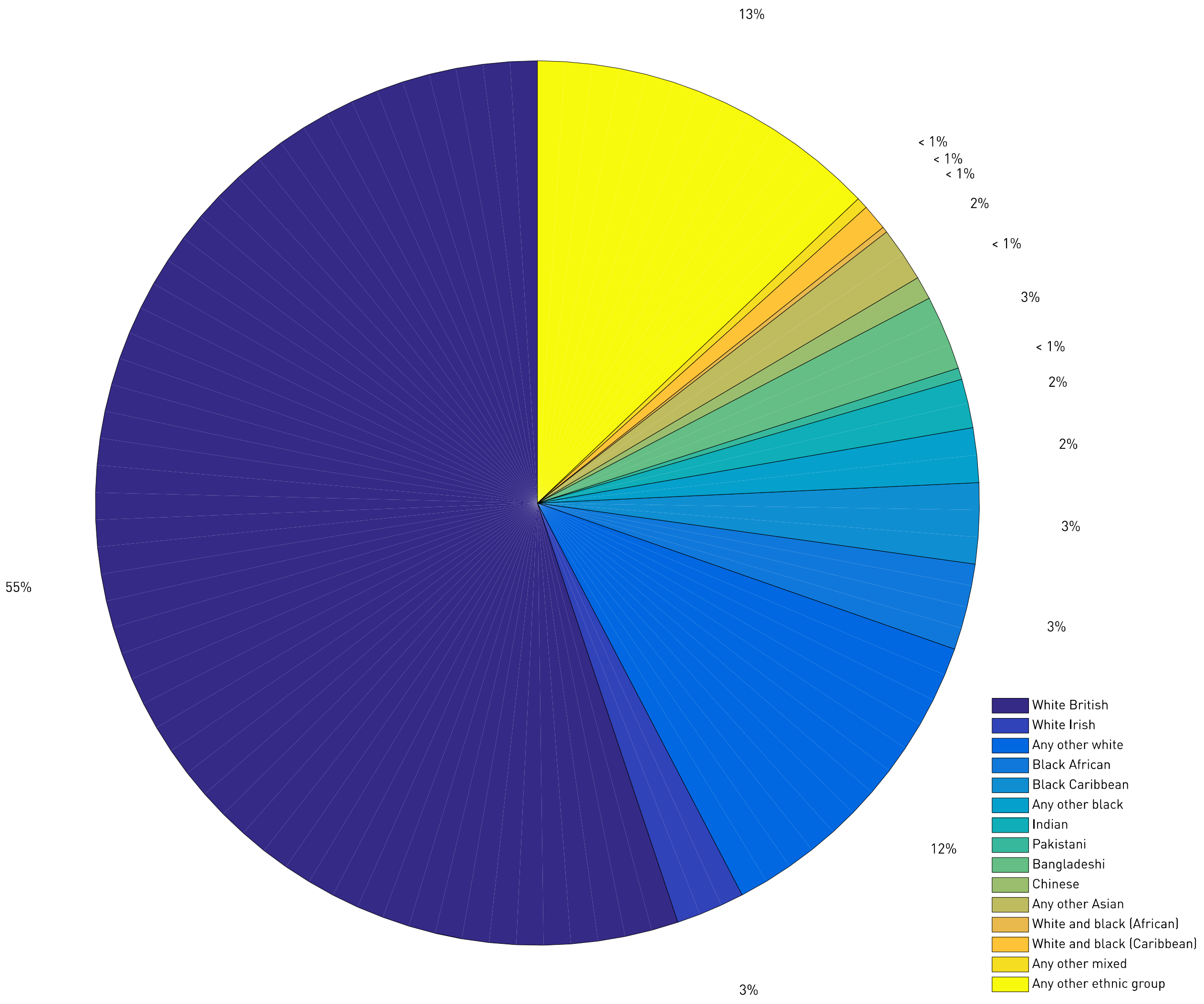

Supplementary Figure 2

The distribution of self-reported patient ethnicity for the 71% of patients in whom it was recorded.

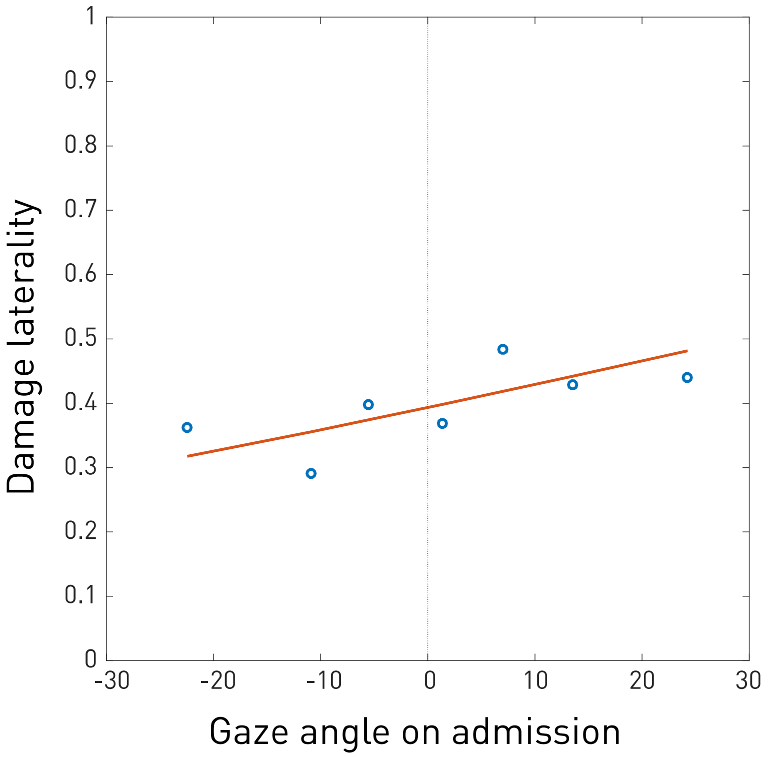

Supplementary Figure 3

Relation between the direction of gaze and the laterality of brain damage at the time of the MRI. For each of seven bins of gaze angle, the mean ratio of the volume of right hemisphere damage to the overall volume of damage is plotted (in blue), with a general linear model maximum likelihood fit of the relation across gaze (in red). The remarkably strong dependence of *admission* gaze on damage laterality shown in Fig. 2 is here much less pronounced, as one would expect if the neural dependents of recovery are more complex.
